# Hyperemesis Gravidarum

**Published:** 1907-11

**Authors:** W. T. Marrs

**Affiliations:** Peoria, Illinois


For the Texas Medical Journal.

### Hyperemesis Gravidarum.

W. T. MARRS, M. D., PEORIA, ILLINOIS.

This is the scientific term to cover the obstinate vomiting of pregnancy. This troublesome, if not to say dangerous condition usually depends upon three factors of causation. These are reflex, toxemic and neurotic. It has been the experience of the writer to observe that the first named is a concomitant of early gestation, while the second occurs more often toward the middle of pregnancy. The neurotic type quite often complicates the other two, and may occur at any time. The neurotic element is more marked in cases of primipara. The writer recently saw a nulliparous woman who in the supposed second month of pregnancy suffered from the reflex type. During three weeks not even water was retained on the stomach and the slight renal excretion was maintained from enemas. The woman was removed to a hospital and all remedial and mechanical measures were vainly tried in the hope of getting the pernicious vomiting under control. As the woman's vitality was fast ebbing away from inanition and the exhaustion of vomiting, we determined, after deliberate counsel, to empty the uterus. This was done by moderate mechanical dilatation and tamponage. The vomiting ceased, and the woman soon became convalescent.

These cases are trying indeed, for the physician is puzzled to form an opinion as to the prognosis, whether he treats the patient on the expectant plan or whether he decides to terminate the pregnancy. In either event the case may go wrong. If radical treatment is to be resorted to, it should not be delayed too long and competent counsel should be secured early. It is well to divide the praise or blame in these cases with at least two other reputable medical men. If the trouble has progressed into the weeks and the pharmacopeia has offered no relief; if nutrient and saline enemas are unavailing and the urine shows an excess of toxines, it is time to get the opinion of other medical men. Before determining upon emptying the uterus it is well, especially in all cases in which there is a reflex element, to paint the cervix with iodine and carbolic acid and to try to correct any malposition that might be present. If all measures fail and the woman's vital powers are failing it is then the duty of the physician to terminate the gestation and give her a chance for her life.

